# MAP4K4 controlled integrin β1 activation and c-Met endocytosis are associated with invasive behavior of medulloblastoma cells

**DOI:** 10.18632/oncotarget.25294

**Published:** 2018-05-01

**Authors:** Dimitra Tripolitsioti, Karthiga Santhana Kumar, Anuja Neve, Jessica Migliavacca, Charles Capdeville, Elisabeth J. Rushing, Min Ma, Noriyuki Kijima, Ashish Sharma, Martin Pruschy, Scott McComb, Michael D. Taylor, Michael A. Grotzer, Martin Baumgartner

**Affiliations:** ^1^ University Children’s Hospital Zürich, Department of Oncology, Children’s Research Center, Zürich, Switzerland; ^2^ Institute of Neuropathology, University Hospital Zürich, Zürich, Switzerland; ^3^ Division of Neurosurgery, The Hospital for Sick Children, University of Toronto, Toronto, ON, Canada; ^4^ Department of Radiation Oncology, University Hospital Zürich, Zürich, Switzerland; ^5^ University Children’s Hospital Zürich, Department of Oncology, Zürich, Switzerland

**Keywords:** medulloblastoma, MAP4K4, integrin β1, c-Met, cell migration and invasion

## Abstract

Local tissue infiltration of Medulloblastoma (MB) tumor cells precedes metastatic disease but little is still known about intrinsic regulation of migration and invasion in these cells.

We found that MAP4K4, a pro-migratory Ser/Thr kinase, is overexpressed in 30% of primary MB tumors and that increased expression is particularly associated with the frequently metastatic SHH β subtype. MAP4K4 is a driver of migration and invasion downstream of c-Met, which is transcriptionally up-regulated in SHH MB. Consistently, depletion of MAP4K4 in MB tumor cells restricts HGF-driven matrix invasion *in vitro* and brain tissue infiltration *ex vivo*. We show that these pro-migratory functions of MAP4K4 involve the activation of the integrin β-1 adhesion receptor and are associated with increased endocytic uptake. The consequent enhanced recycling of c-Met caused by MAP4K4 results in the accumulation of activated c-Met in cytosolic vesicles, which is required for sustained signaling and downstream pathway activation.

The parallel increase of c-Met and MAP4K4 expression in SHH MB could predict an increased potential of these tumors to infiltrate brain tissue and cause metastatic disease. Molecular targeting of the underlying accelerated endocytosis and receptor recycling could represent a novel approach to block pro-migratory effector functions of MAP4K4 in metastatic cancers.

## INTRODUCTION

Medulloblastoma (MB), the most common malignant brain tumor in childhood, arises in the cerebellum, locally infiltrates and has the propensity for leptomeningeal spread through the cerebrospinal fluid (CSF). MB is molecularly classified into the subgroups WNT (Wingless), SHH (Sonic hedgehog) p53-wild-type and SHH p53-mutant and groups 3 and 4 [1]. Large-scale, genome-wide DNA methylation and gene expression analyses combined with copy number aberrations and clinical features have recently allowed to further refine the four subgroup classification of MB [2] into a total of 12 subtypes [3, 4]. Of particular significance is the finding that SHH β subtype tumors are frequently metastatic, a feature that has so far not been attributed to the SHH subgroup and that is mechanistically not understood [4]. With aggressive treatment modalities, survival rates across all MB subgroups of approximately 70% can be reached [5]. However, metastatic and recurrent MB remains clinically very challenging with high mortality rates.

Growth factor-driven cancer cell infiltration relies on elevated vesicle trafficking for membrane receptor turnover and extracellular matrix (ECM) remodeling [6, 7]. Endocytic turnover of receptor tyrosine kinases (RTKs) for example such as c-Met [8], FGFR [9] or EGFR [10] determines strength, specificity and duration of signaling. In addition, the redistribution of the integrin adhesion receptors through endocytic recycling is also critical for cell migration. Integrin engagement, activation and recycling is required for the formation of invasive structures such as filopodia [11, 12], which direct cell migration in complex tissue environments. Indeed, the aberrant recycling, activation and/or cell surface presentation of integrins can enhance the invasive behavior of cancer cells [13–15]. Consequently, ß1 integrin trafficking has been identified as a novel therapeutic target for anti-metastatic therapy [16]. However, how growth factor signaling is coupled to receptor trafficking is incompletely understood in general and its potential implication in pediatric brain cancers has not been investigated.

One kinase linking both integrin activation and RTK signal transmission towards migration and invasion is the serine/threonine protein kinase MAP4K4 [17]. Genetic interference and gene expression analyses have implicated MAP4K4 activity in a plethora of cellular functions relevant for physiological and pathophysiological processes, including organ development, systemic inflammation, metabolic disorders and in particular cancer [17, 18]. MAP4K4 functions downstream of the c-Met receptor tyrosine kinase in MB cells to control migration and invasion [19]. Moreover, high expression of MAP4K4 in lung adenocarcinoma is sufficient to activate the MAP kinase ERK for cancer growth and dissemination [20].

Here, we investigated the relationship between the increased expression of MAP4K4 and the biology of migration and invasion in MB. We addressed how actin-targeting functions of MAP4K4 are coupled to migration and invasion and specifically focused on the regulation of receptor turnover in response to growth factor stimulation and matrix adhesion.

## RESULTS

### The Ser/Thr kinase MAP4K4 is overexpressed in primary medulloblastoma

**Figure 1:**
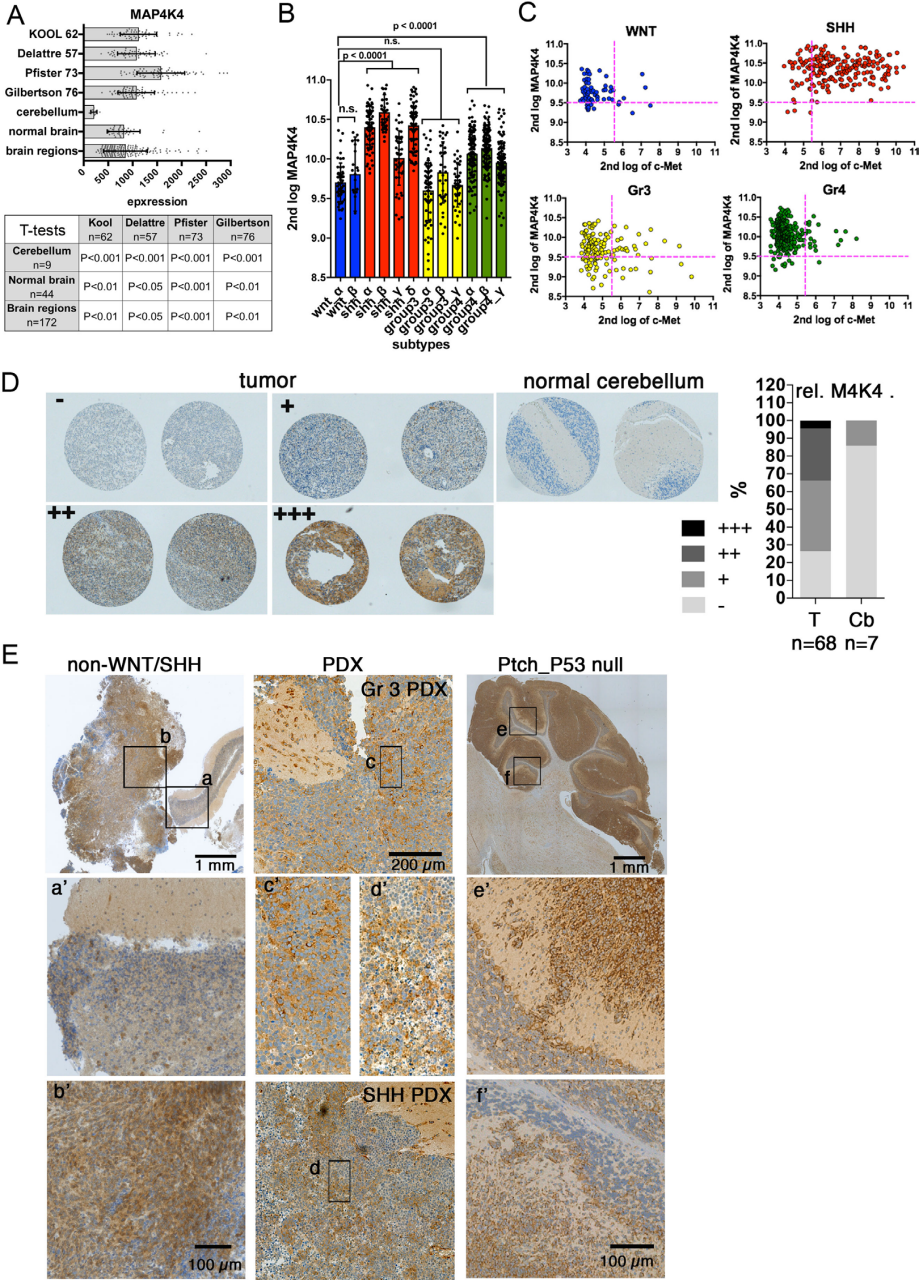
MAP4K4 is overexpressed in MB tumors **(A)** MAP4K4 mRNA U133P affymetrix gene chip micro array expression levels in normal tissue samples versus MB tumors. X axis shows absolute intensity values of hybridization reads. Same chip technology and internal controls used for all sample groups. Table shows p values of t-tests of the comparisons of the samples indicated in left column and top row. **(B)** MAP4K4 expression analysis in the twelve MB subtypes using data derived from the affymetrix human gene 1.1 ST array profiling of the Cavalli gene set of 763 primary MB samples. **(C)** Comparative MAP4K4 and c-Met expression analysis in the four MB subgroups using data derived from the affymetrix human gene 1.1 ST array profiling of the Cavalli gene set of 763 primary MB samples. **(D)** MAP4K4 expression in MB tumor tissue microarray of MB tumor (T) and cerebellum tissue (Cb) samples and H score quantification. **(E)** Anti-MAP4K4 IHC analysis in a non-WNT/non-SHH MB primary tumor, a G3 MB PDX and a Ptch/P53 null SHH model of MB. a’ – f’ are 4x magnifications of boxed areas. a’ is healthy cerebellar tissue, and b’ is tumor tissue. c’ and d’ are different zones in same tumor. e’ and f’ show MAP4K4-positive neoplastic cells infiltrating the granular layer.

MAP4K4 expression is associated with tumor progression and metastasis in different solid tumors [20–24]. MAP4K4 mRNA expression is also increased in MB samples derived from several patient cohorts compared to normal cerebellum (cb) or normal brain (Figure 1A). The analysis of affymetrix human gene 1.1 ST array profiling of a recently assembled set of 763 primary MB samples used for subtype identification revealed increased MAP4K4 expression in the SHH subgroup, with particular enrichment in the SHH-α, β and δ subtypes [4] (Figure 1B). The majority of high MAP4K4 SHH tumors also express high levels of the c-Met receptor tyrosine kinase (Figure 1C), which is also particularly enriched in all four SHH subtypes (Supplementary Figure 1A). To gain insight in MAP4K4 protein expression in human MB tumor tissue, we scored MAP4K4 expression levels by immunohistochemistry (IHC) into negative, low, moderate and high in a MB tissue micro array (TMA) containing 68 human tumor samples and 7 cb controls. We observed increased MAP4K4 in MB tissue compared to adjacent cb, with some tumors showing very high levels of MAP4K4 (Figure 1D). IHC also detected high levels of MAP4K4 protein expression in a non-WNT/SHH MB, moderate levels in a group 3 patient-derived xenograft (PDX) and very high expression levels in a Ptch/P53 null genetic mouse model (Figure 1E). Thus, MAP4K4 expression is increased in MB compared to healthy cerebellum and increased levels can be detected both at the mRNA and the protein levels.

### MAP4K4 promotes clonogenicity in SHH MB cells

**Figure 2:**
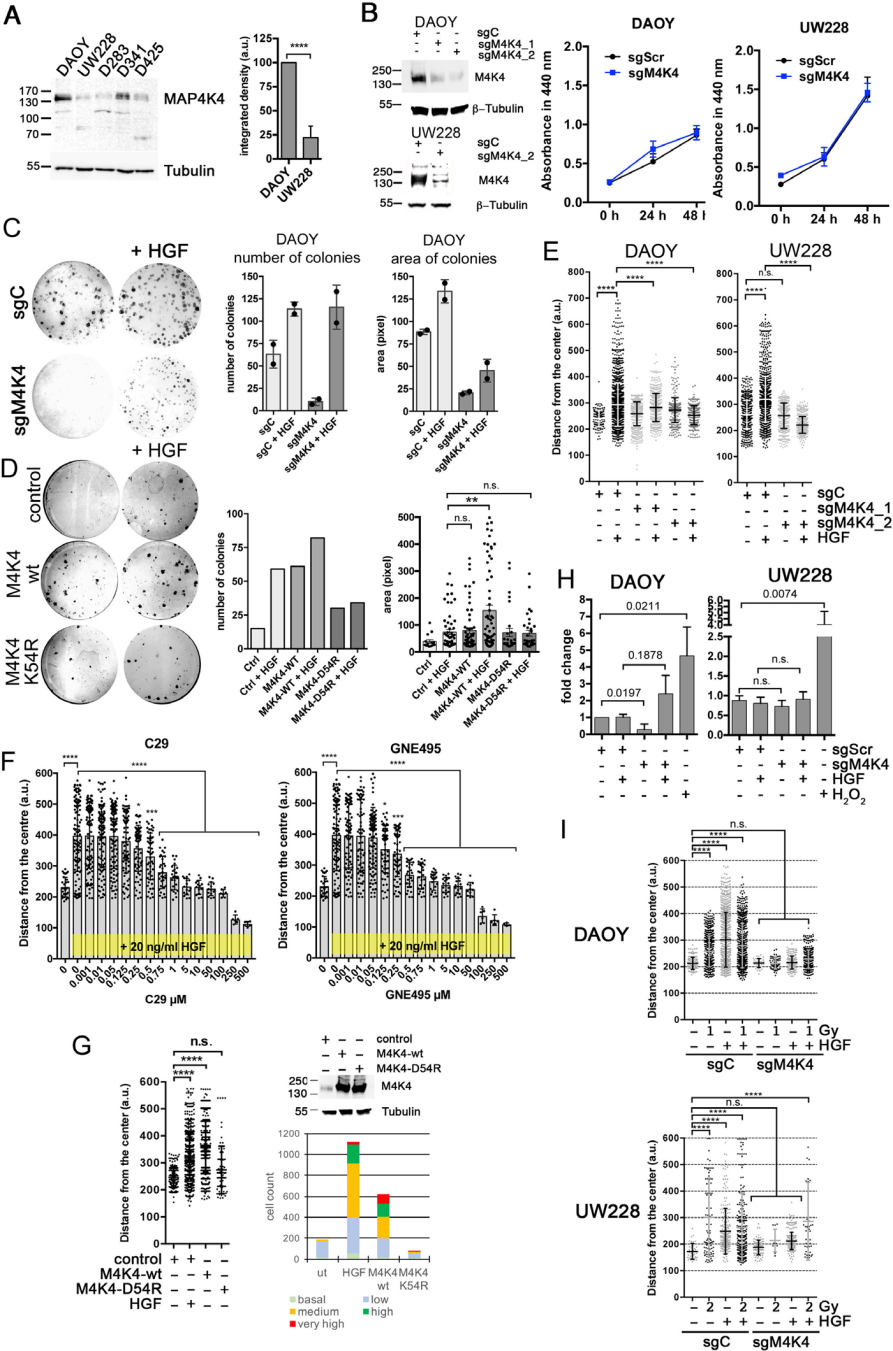
MAP4K4 contributes to oncogenic phenotype **(A)** IBanalysis of MAP4K4 protein expression in various MB cell lines and comparative quantification of MAP4K4 protein expression in DAOY and UW228 cells (n=3 i.e.). **(B)** left: IB analysis of knock-down efficiency of sgM4K4 in DAOY and UW228 cells. Right: WST assay of DAOY and UW228 cells grown under normal growth conditions (triplicate measurements). **(C)** Clonogenic assay of DAOY cells and quantification of mean colony number and area (n=2 i.e.). **(D)** Clonogenic assay of DAOY control cells or DAOY cells expressing either M4K4-wt or M4K4-K54R and quantification of mean colony number and area (n=1). **(E)** SIA of cells with CRISPR/CAS9-mediated knock-down of MAP4K4 -/+ HGF. **(F)** Dose-response analysis using SIA of DAOY cells -/+ C29 or -/+ GNE-495 in the presence of 20 ng/ml HGF. Pooled data of two independent experiments are shown. ^*^p < 0.05, ^**^p < 0.01, ^***^p < 0.001, ^****^p < 0.0001 (one-way Anova). **(G)** SIA with DAOY control, or DAOY ectopically expressing either MAP4K4 wild-type (wt), or MAP4K4 kinase-dead mutant (K54R) and frequency distribution for each condition (basal: 100 – 200, low: 200 – 300, intermediate: 300 – 400, high: 400 – 500, very high: 500+). IB shows MAP4K4 expression in control and overexpressing cells. ^*^p < 0.05, ^**^p < 0.01, ^***^p < 0.001, ^****^p < 0.0001 (one-way Anova). **(H)** CellTox Green cytotoxicity assay measured in detached tumor cell spheroids (n=3 i.e.). **(I)** Representative dot plots of SIA of DAOY and UW228 cells (sgControl and sgMAP4K4_2) exposed to one (DAOY) or two (UW228) Gray (Gy) photon irradiation after embedding in collagen. SIA was performed immediately after irradiation -/+ 20 ng/ml HGF as indicated. ^*^p < 0.05, ^**^p < 0.01, ^***^p < 0.001, ^****^p < 0.0001 (one-way Anova).

We next assessed MAP4K4 mRNA (Supplementary Figure 1B) and protein (Figure 2A) expression levels in established laboratory or PDX SHH or G3 lines to identify a model cell line for further molecular analysis of MAP4K4 function. Variable expression levels were seen in all MB lines. Significantly higher MAP4K4 protein expression was detected in DAOY compared to UW228 cells. Although considered SHH MB lines [25], methylation and expression profiling revealed differences between DAOY and UW228 and primary SHH MB subgroup tumors. However, recent comparative proteomic analysis identified a number of signature proteins and revealed enrichment of key components of SHH and WNT signaling pathways in DAOY and UW228, respectively [26]. We therefore compared expression levels of the five NCBI-listed MAP4Ks (MAP4K1-5) in DAOY cells, and found MAP4K4 expression levels to be the highest in these cells (Supplementary Figure 1C). In both the DAOY and the UW228 cells, MAP4K4 protein was detected in and around the nucleus and in lamellipodia (Supplementary Figure 1D). Using variant-specific siRNAs, we found that only the depletion of MAP4K4 or MAP4K5 prevented hepatocyte growth factor (HGF)-induced single cell migration we previously described in DAOY cells [19] (Supplementary Figure 1E). Based on its comparatively high expression, we considered MAP4K4 the predominant MAP4 kinase in the HGF-c-Met pathway in DAOY cells. Using CRISPR/CAS9 we generated DAOY and UW228 cell lines with greatly reduced MAP4K4 expression (Figure 2B). Under normal tissue culture conditions, the sgMAP4K4_2 (sgM4K4) cells displayed no decreased viability compared to sgControl (sgC) cells (Figure 2B). We next tested the impact of MAP4K4 depletion on colony forming activity and clonal growth in DAOY cells (Figure 2C). Interestingly, the number and the size of colonies were markedly reduced in MAP4K4-depleted cells. Supplementing the normal growth medium with HGF rescued colony forming activity but not clonal growth in DAOY cells (Figure 2C). Overexpression of MAP4K4-wt but not of kinase-dead MAP4K4-K54R [21] induced colony forming activity and clonal growth comparable to HGF treated control cells (Figure 2D). Although MAP4K4 depletion also moderately reduced the number of colonies in UW228 cells, we measured no increase in colony-forming activity or clonal growth in the presence of HGF (not shown). However, closer inspection of the cells indicated that HGF stimulation caused cell scattering and morphological alterations in UW228, which in part explained the failure to detect colonies by crystal violet staining. These data thus indicate that MAP4K4 function and HGF signaling are important for attributing clonogenic properties to the DAOY cells.

### MAP4K4 is required for migration and invasion

To determine whether reduction of MAP4K4 expression in MB cells is sufficient to impede HGF-induced collagen I (collagen I) invasion, we quantified cell migration and invasion using the spheroid invasion assay (SIA [27], Supplementary Figure 2A). MAP4K4 depletion ablated HGF-induced migration in both DAOY and UW228 cells (Figure 2E). Tetracycline-inducible short hairpin RNA targeting of MAP4K4 mRNA [19], also significantly impaired HGF-, EGF- and IGF-induced collagen I invasion but did only moderately affect dissemination induced by bFGF (Supplementary Figure 2B). MAP4K4 kinase inhibition with compound 29 (C29) [28] or the second generation MAP4K4 inhibitor GNE-495 [29] prevented HGF-induced collagen I invasion (Figure 2F). Complete repression of HGF-induce collagen I invasion was achieved for C29 and GNE-495 by 5 and 1 μM, respectively. The over-expression of the wt but not of kinase-dead MAP4K4 triggered collagen I invasion in the absence of HGF similar to control cells in the presence of HGF (Figure 2G). As MAP4K4 depletion may affect viability of the cells grown under three dimensional (3D) conditions and detached of substrate adhesion, we measured CellTox Green uptake of DAOY and UW228 cells grown as non-embedded spheroids. CellTox Green uptake was moderately increased in sgM4K4 DAOY cells stimulated with HGF (Figure 2H), indicating that MAP4K4 depletion combined with GF stimulation in the absence of substrate adhesion could cause loss of viability.

Sub-lethal doses of radiation can induce pro-metastatic phenotypes of tumor cells through extrinsic [30, 31] or intrinsic alterations [32–35]. To explore whether MAP4K4 could also be involved in radiation-induced migration, we determined the impact of irradiation (IR) on collagen I invasion. We found that IR doses of 1 and 2 Gy increased collagen I invasion of sgC DAOY and sgC UW228 cells, respectively (Figure 2I). IR induction of collagen I invasion is more modest compared to HGF and we observed no synergistic effects when irradiation was combined with HGF treatment. Depletion of MAP4K4 significantly reduced IR-induced collagen I invasion both in the absence or presence of HGF. Thus, MAP4K4 controls a molecular mechanism that is required for migration and invasion control but that is not strictly dependent on a specific extracellular cue.

### MAP4K4 is necessary for brain tissue infiltration *ex vivo*

**Figure 3:**
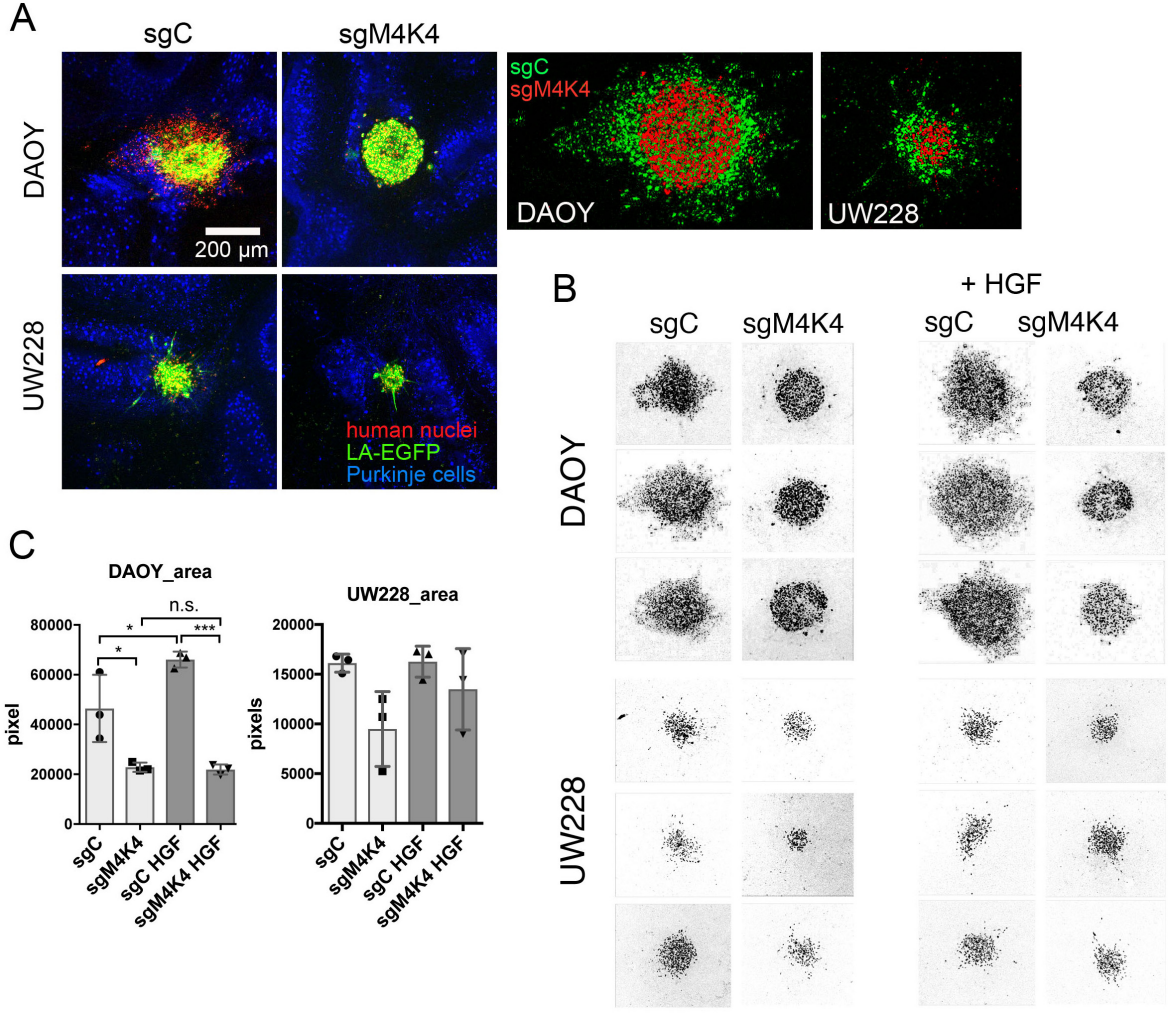
MAP4K4 promotes brain tissue infiltration **(A)** Left:Organotypic cerebellum slice culture with DAOY sgCtrl or DAOY sgMAP4K4_2 spheroids implanted. 10x image acquisition of representative slices five days after implantation. Red: Human nuclei, green: Lifeact-EGFP, blue: Calbindin. Right: overlay of human nuclei staining of sgC (green) and sgM4K4 (red) spheroids **(B)** Inverted grey scale images of human nuclei staining for comparison of tumor cell expansion in slices. **(C)** Quantification of areas of human nuclei staining shown in B.

MB tumor cells grow on and invade heterologous brain tissue *ex vivo* [36]. To test whether depletion of MAP4K4 prevented this infiltration process, we implanted tumor cell spheroids on the surface of cerebellum slices and monitored the dissemination process after five days of incubation. We found that sgC cells began to infiltrate the brain tissue and to disseminate from the tumor spheroid. Depletion of MAP4K4 caused rounded cell morphology and markedly (DAOY) or moderately (UW228) reduced brain tissue infiltration (Figure 3A). Furthermore, MAP4K4 depletion diminished focal, F-actin-positive invasion structures in DAOY cells (Supplementary Figure 2C). The quantification of the area covered by the expanding tumor cells using the anti-human nuclei signal (Figure 3B) revealed a further increase in expansion in HGF-stimulated DAOY cells, which was abrogated in MAP4K4-depleted cells. Consistent with the lower expression of MAP4K4 in UW228 cells, the effect of MAP4K4 depletion on tissue infiltration in UW228 cells was less marked (Figure 3B, 3C). Hence, MAP4K4 function is necessary for brain tissue infiltration and the efficacy of the process may depend on the level of MAP4K4 in the tumor cells.

### HGF-induced actin dynamics and endocytic activity require active MAP4K4

**Figure 4:**
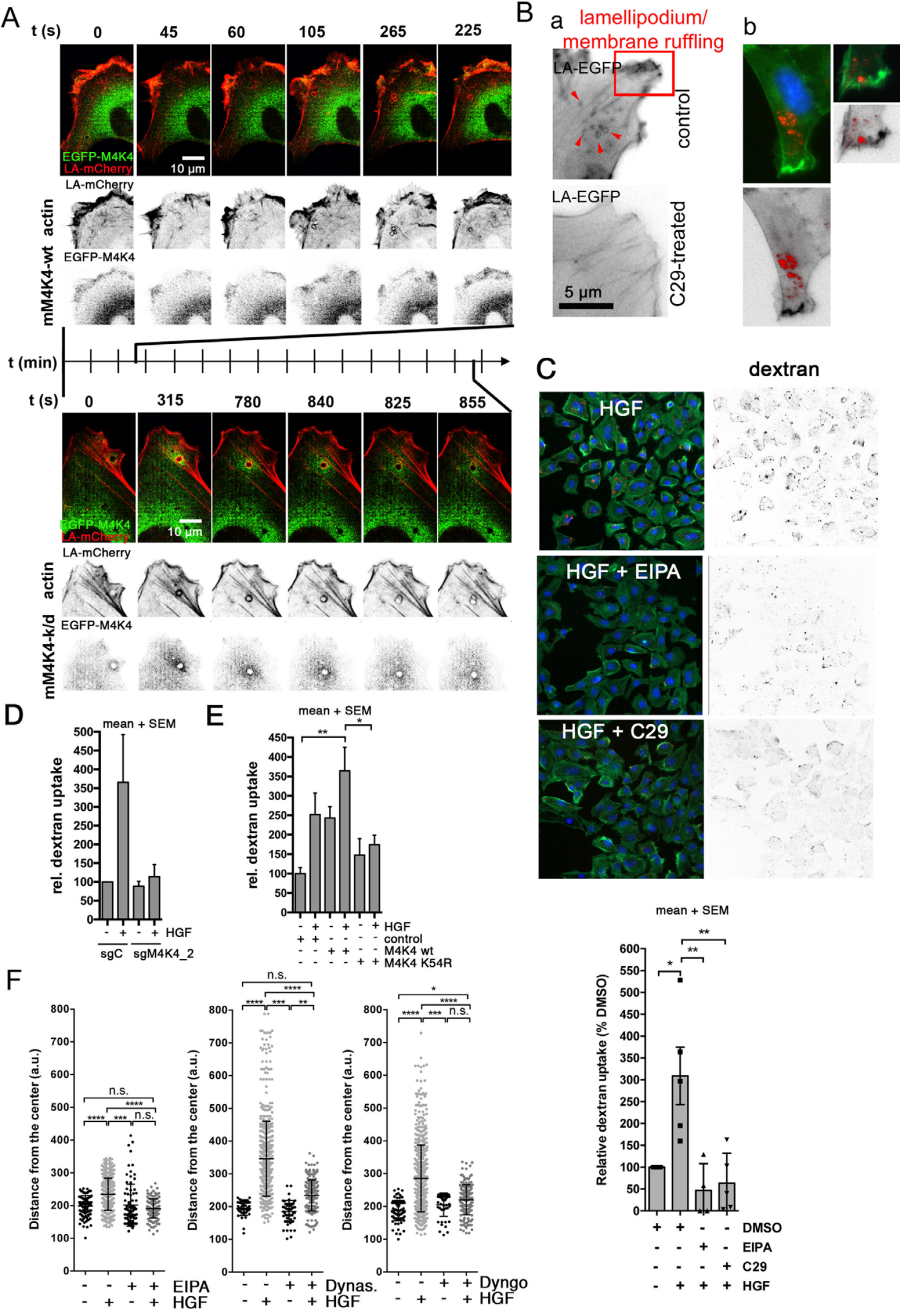
MAP4K4 promotes endocytic uptake **(A)** Still images of time lapse movies of DAOY cells transfected with vectors expressing lifeact-mCherry and either wild-type or kinase-dead (k/d) EGFP-Nck-interacting kinase NIK (murine MAP4K4, mM4K4-wt and mM4K4D152N). Inverted grey scale images show actin and EGFP-MAP4K4. **(B)** Left: LA-EGFP expressing DAOY cells treated with DMSO or MAP4K4 inhibitor C29 (2.5 μM, 48h), LA-EGFP in grey scale. Arrows point to trafficking vesicles. Right: IFA analysis of cells exposed to 70 kDa DS red dextran for 30 min. **(C)** 70 kDa DS red dextran uptake analysis in DAOY cells. Upper: IFA showing representative images of DAOY cells 30 min after exposure to dextran + HGF and -/+ EIPA (25 μM) or C29 (2.5 μM). Red: Dextran, green: F-actin, blue: DNA. Lower: quantification of HGF-dependent dextran uptake and impact of EIPA or C29 treatment 30 min after exposure to dextran (n=3 i.e.). **(D)** Quantification of HGF-induced 70 kDa DS red dextran uptake in sgC and sgM4K4 DAOY cells (n=2 i.e.). **(E)** Quantification of HGF-induced 70 kDa DS red dextran uptake in control DAOY or DAOY cells overexpressing MAP4K4-wt or MAP4K4-K54R (kinase-dead, (n=3 i.e.)). **(F)** SIA of HGF-induced collagen I invasion -/+ EIPA (25 μM) or Dynasore (20 μM) or Dyngo (5 μM) treatment (means ± SEM, ^*^p < 0.05, ^**^p < 0.01, ^***^p < 0.001, ^****^p < 0.0001 (one-way Anova).

MAP4K4 (Supplementary Figure 1D) and EGFP-Nck-interacting kinase NIK (murine ortholog of human MAP4K4 (Figure 4A, Supplementary Movie 1)) co-localized with dynamic F-actin at the leading edge of migrating cells. Expression of EGFP-NIK-k/d (D152N), which associated with F-actin-decorated vesicles, reduced membrane ruffling and the speed of vesicle trafficking (Figure 4A, Supplementary Movie 2). Consistently, C29 treatment completely abrogated ruffling and the emergence of F-actin coated vesicles (Figure 4Ba). The macropinocytosis marker 70 kDa dextran was taken up by the cells (Figure 4Bb). This uptake was enhanced by HGF (Figure 4C) or EGF (not shown), suggesting GF induction of endocytic activity in the tumor cells. Pre-treatment with C29 abrogated HGF-induced dextran up-take to a similar level as observed after the pretreatment with the macropinocytosis inhibitor ethylisopropylamiloride (EIPA) (Figure 4C). Depletion of MAP4K4 impaired HGF-induced dextran endocytosis (Figure 4D), whereas the overexpression of MAP4K4-wt increased endocytic activity to a level similar to HGF-stimulated cells (Figure 4E). Blockade of endocytosis with EIPA, or Dynasore or Dyngo abrogated HGF-induced collagen I invasion (Figure 4F). These data showed that MAP4K4 function accelerated vesicle trafficking and endocytosis, and that latter is necessary to enable HGF-induced collagen I invasion.

### MAP4K4 promotes integrin activation

**Figure 5:**
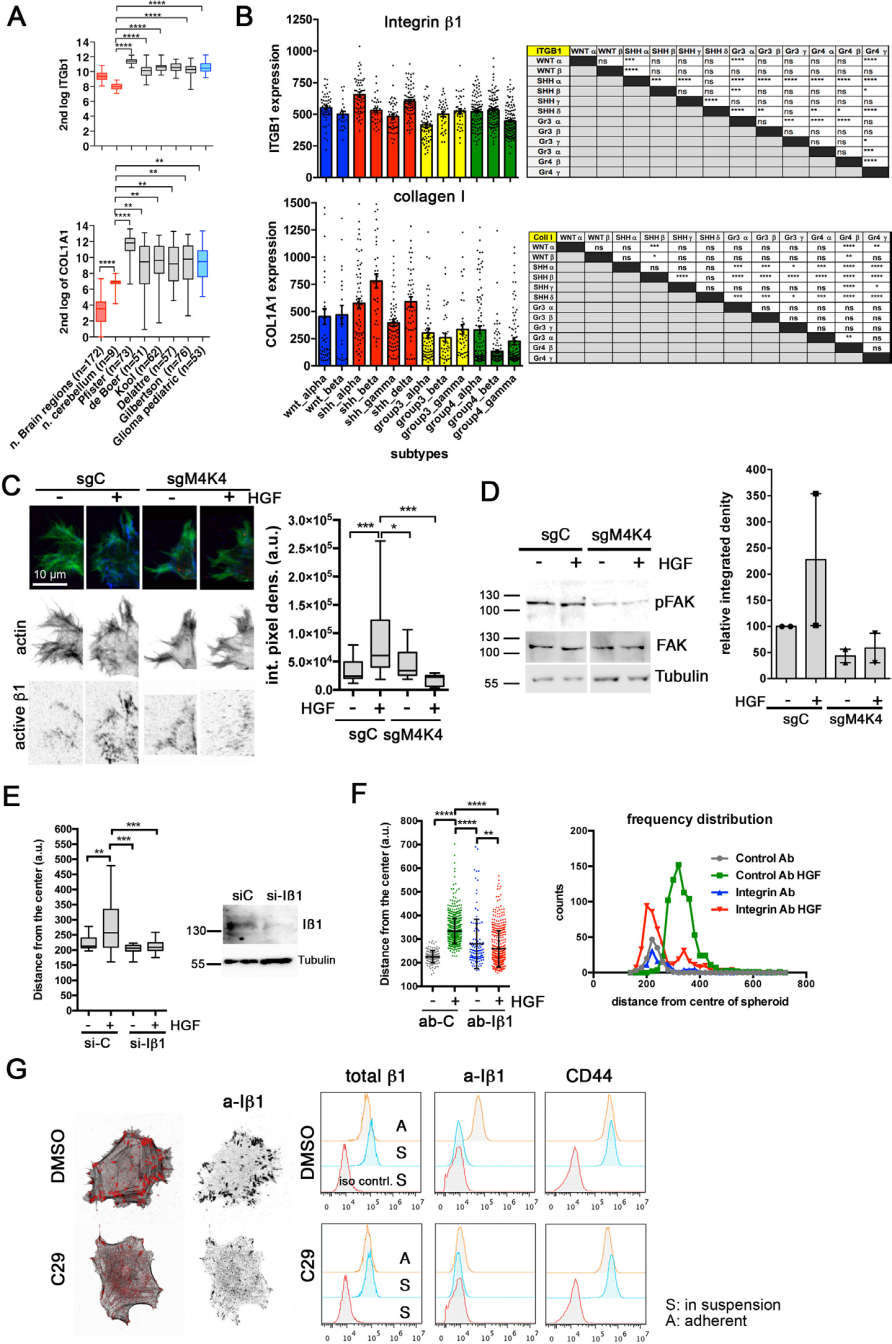
MAP4K4 promotes integrin activation during collagen I invasion **(A)** ITGb1 and COL1A1 mRNA U133P affymetrix gene chip micro array expression levels in normal tissue samples versus MB tumors. Y axis shows absolute intensity values of hybridization reads. Same chip technology and internal controls used for all sample groups. **(B)** Left: COL1A1 and ITGb1 expression analysis using data derived from the affymetrix human gene 1.1 ST array profiling of a set of 763 primary MB samples.Right: Tables showing one-way ANOVA repeated measures test using Bonferroni's Multiple Comparison. P-Values < 0.05 were considered significant (^*^ p ≤ 0.05, ^**^ p ≤ 0.01, ^***^ p ≤ 0.001, ^****^ p ≤ 0.0001). **(C)** Left: representative images of a-Iß1 in lamellipodia of sgScr (sgC) or sgMAP4K4_2 DAOY cells invading collagen I matrix. La-EGFP is in green, a-Iß1 in blue. Inverted grey scale images are shown for better visualization of signals. Right: quantification of integrated pixel density of a-Iß1 signal in lamellipodia. **(D)** IB and quantification of FAK phosphorylation in collagen I-embedded DAOY sgC and sgMAP4K4_2 cells -/+ HGF stimulation for 18 h. Mean and SEM of two independent experiments are shown. **(E)** Left: SIA of control or Iß1-depleted cells -/+ HGF. Right: IB of Iß1 after siRNA-mediated depletion in DAOY cells. **(F)** Dot plot and frequency distribution of blocking Iß1 or control ab effect on collagen I invasion in SIA -/+ HGF. **(G)** Left: Confocal IFA analysis depicting a-Iβ1 at basal side of cells. Right:Histograms of FACS comparison of Iß1, a-Iß1 and CD44. expression in suspension (S) or after 15 min collagen I adhesion (A) in DAOY cells treated either with DMSO or C29.

Integrin adhesion receptors and their coordinated turnover through the endocytic machinery control migration and invasion [6, 37]. Expression of collagen I (COL1A1), the ligand of the ß1 integrin adhesion receptor (ITGb1) is increased in MB compared to normal brain regions or normal cerebellum (Figure 5A). Subtype analysis indicated that COL1A1 expression is higher in SHH-β and SHH-δ compared to the other subtypes (Figure 5B), which both displayed also higher MAP4K4 expression (Figure 1B). Collagen I invasion triggered by HGF is associated with activated ß1 integrin (a-Iß1) enrichment in lamellipodia at the leading edge of invading MB cells (Figure 5C, Supplementary Figure 3). This HGF-dependent a-Iß1 enrichment was abrogated in MAP4K4-depleted cells. Reduced a-Iß1 in collagen I-embedded MAP4K4-depleted cells was accompanied by a reduction of FAK activation, which could not be rescued by HGF treatment (Figure 5D). Pharmacological inhibition of MAP4K4 by C29 or GNE-495 prevented HGF-induction of pFAK in collagen-embedded cells as well (Supplementary Figure 4). Iß1 is necessary for invasion of MB cells as depletion (Figure 5E) or antibody-mediated blocking of Iß1 (Figure 5F) caused a significant reduction in collagen I invasion. MB cell adhesion to collagen I-coated surfaces caused a-Iß1 accumulation at the basal side of the cells (Figure 5G, left) and led to a global increase in surface a-Iß1 within 15 min, compared to cells in suspension (Figure 5G, right). Pre-treatment of the cells with C29 prevented increased basal and surface a-Iß1 in response to collagen binding. C29 pre-treatment did not impact Iß1 or CD44 surface expression, suggesting that reduced surface a-Iß1 after C29 treatment is not a consequence of increased a-Iß1 internalization.

These findings indicate that MAP4K4 pro-migratory activity could in part be mediated through its function towards Iß1 and possibly through the downstream Iß1 effector FAK, which is an established promoter of proliferation, migration and invasion in MB [38].

### MAP4K4 controls c-Met function

**Figure 6:**
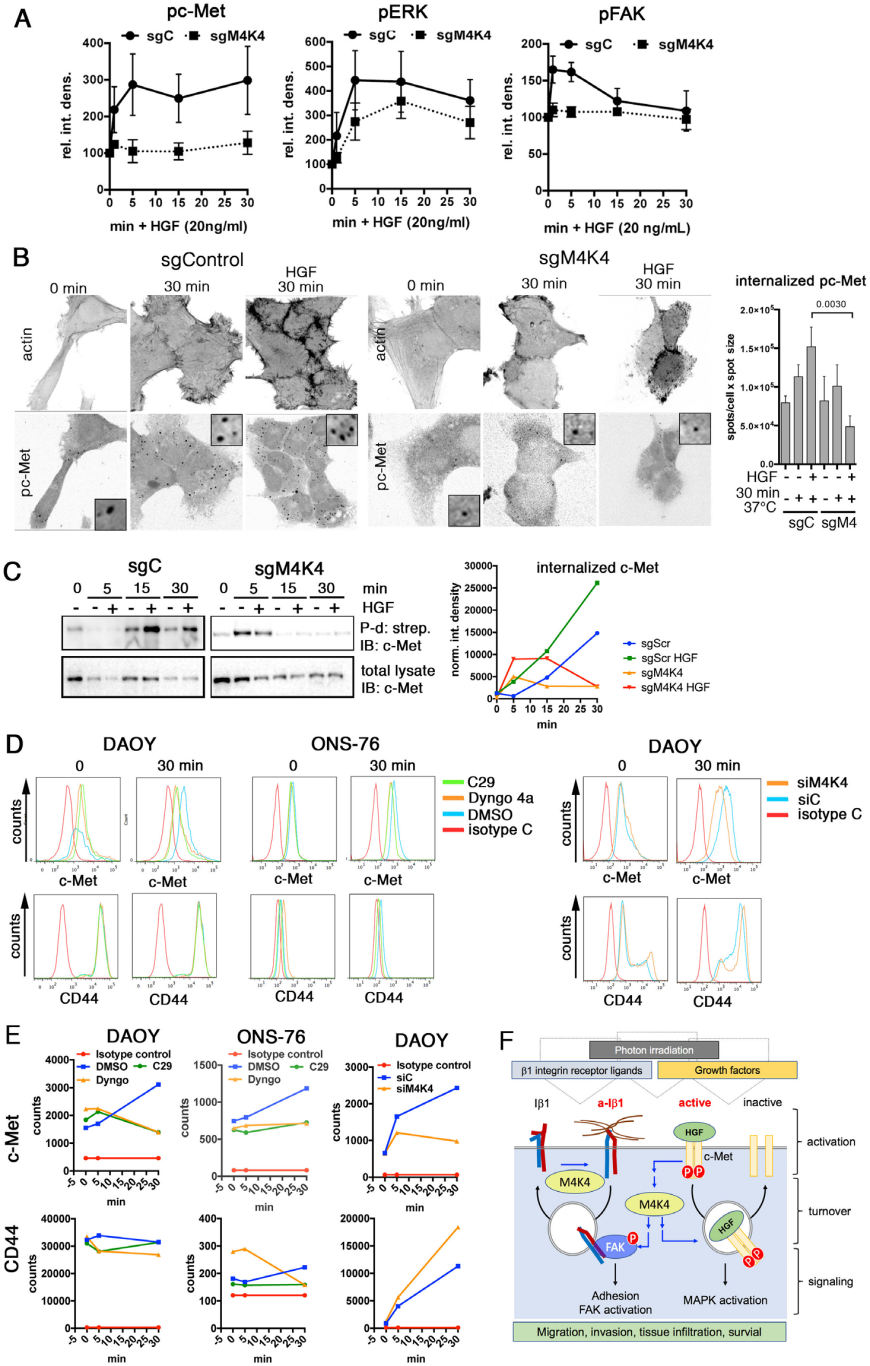
MAP4K4 promotes activation and internalization of c-Met **(A)** Quantification of IB analysis of c-Met, Erk1/2 and FAK phosphorylation in response to HGF stimulation in sgC or sgMAP4K4_2 cells. XY line plots of mean integrated pixel densities and SEM of phospho bands from 3 experiments against time in min after HGF stimulation. **(B)** Left: Confocal IFA of sgC or sgMAP4K4_2 cells adhering to poly-L-lysine. pc-Met was detected before (0 min) and after raising temperature from 0° to 37°C -/+ HGF. Insets are 4x magnifications. Right: bar diagram depicting quantity of internalized pc-Met (number of spots per cell x spot size (n=13 cells, mean and SD). **(C)** Left: IB of internalized, biotinylated c-Met and total c-Met in sgC or sgMAP4K4_2 cells -/+ HGF for the times indicated. Right: Integrated pixel densities of internalized biotinylated c-Met relative to total c-Met. **(D)** FACS analysis of c-Met and CD44 surface expression on DAOY and ONS-76 cells without and after 30 min HGF stimulation and compound or siRNA treatments as indicated. **(E)** Median fluorescence intensities of c-Met and CD44 plotted against time of HGF stimulation from FACS analyses shown in D). **(F)** Scheme depicting MAP4K4 control of integrin α5ß1 and c-Met receptor activation and turnover for establishment of pro-metastatic phenotype.

FAK cooperates with c-Met in MB [38], and c-Met is an established promoter of migration and invasion in MB cells [19, 39]. c-Met is endocytosed upon HGF binding and c-Met signaling from endocytic vesicles mediates growth, survival and cell migration [8]. Blocking endocytosis with Dyngo 4a (Supplementary Figure 5A) or depletion of MAP4K4 (Figure 6A, Supplementary Figure 5B) reduced HGF-dependent c-Met and ERK activation and repressed FAK phosphorylation. This MAP4K4-dependent regulation of c-Met function is specific for DAOY cells and was not observed in UW228 cells (Supplementary Figure 5C). HGF stimulation led to the Dyngo 4a-sensitive accumulation of phospho c-Met (pc-Met) on vesicular structures in the cytoplasm of DAOY cells (Supplementary Figure 5D). The concomitant increase in cortical F-actin and the appearance of pc-Met-decorated vesicles in the cytosol was MAP4K4-dependent (Figure 6B). These data suggested that activated pc-Met traffics through intracellular compartments and either activation or internalization of c-Met requires MAP4K4. To test whether internalization could be affected, we biotinylated surface proteins and measured the abundance of internalized c-Met-biotin in response to HGF stimulation. Raising the temperature from 0 to 37°C caused intracellular c-Met-biotin levels to increase within 30 min (Figure 6C). HGF stimulation further enhanced this effect, whereas depletion of MAP4K4 prevented both temperature shift- and HGF-induced c-Met internalization. To test whether HGF stimulation could affect c-Met recycling and its expression at the plasma membrane, we compared the levels of cell surface c-Met by flow cytometry in unstimulated cells and in cells stimulated with HGF for 30 min. We found that HGF stimulation caused increased c-Met surface expression (Figure 6D, 6E). Increased surface c-Met in HGF stimulated cells required endocytic trafficking as it was prevented by the pre-treatment with Dyngo 4a. Inhibition of MAP4K4 also abrogated increased surface expression of c-Met after HGF stimulation (Figure 6E), indicating that MAP4K4 contribution to HGF-c-Met signaling could in part be mediated through its potential to increase internalization and recycling of c-Met (Figure 6F).

## DISCUSSION

We found that the Ser/Thr kinase MAP4K4 is over-expressed in MB compared to healthy cerebellum tissue. This over-expression promotes migratory and invasive capabilities of MB tumor cells in response to HGF-c-Met stimulation or photon irradiation. We furthermore show that increased MAP4K4 expression and function are associated with enhanced endocytic activity and that this activity of MAP4K4 promotes turnover and activation of the Iß1 adhesion receptor and of the c-Met receptor tyrosine kinase. This study thus revealed a novel mechanistic link between MAP4K4 control of receptor turnover and activation and tissue infiltration in MB (Figure 6F). Furthermore, it identified a potentially druggable mechanism to restrict tumor cell migration and invasion by interfering with the endocytic processing of adhesion and growth factor signaling.

Increased Iß1 and c-Met activation and turnover have been linked to cancer progression and metastasis [40, 41], by specifically enabling motile and invasive capabilities. Our data indicate that MAP4K4 couples c-Met signaling to integrin function in invasive protrusions, and that it is either involved in inside-out signaling for integrin activation or in the trafficking of Iß1 to sites of focal contacts. Former is supported by the observation that MAP4K4 inhibition prevents surface a-Iß1, latter is indicated by our finding that a-Iß1 is reduced at basal adhesions sites after MAP4K4 inhibition. This contrasts with earlier studies that found that MAP4K4 causes focal adhesions (FA) dissolution by negative regulation of Iß1 through moesin-mediated competition for talin binding [42], or through enhanced endocytosis by MAP4K4-activated Arf6 activation via IQSEC1 [43]. However, increased FA dissolution is coupled to increased FA assembly [43], indicating that MAP4K4 does not inactivate Iß1 per se but accelerates its turnover, which is necessary for cell migration. Indeed, MAP4K4 was found on a-Iß1-positive endosomes, which suppress anoikis and promote metastasis through FAK activation [44]. In MB cells invading collagen gels, we observed increased a-Iß1 in HGF-stimulated cells at the invasion fronts, where Iß1 turnover is important for protrusion extension during growth factor-induced tumor cell migration. The failure of MAP4K4-depleted cells to activate Iß1 in these critical structures and the repression of Iß1 activation upon collagen binding in MB cells treated with the MAP4K4 inhibitor C29 indicates that FA dissolution in endothelial cells [42] and keratinocytes [43] could also be the consequence of MAP4K4 activation of recycling Iß1. The parallel MAP4K4-dependent activation of FAK that we observed in MB indicates that MAP4K4 functionally links Iß1 to adhesion-induced FAK activation and collagen I invasion.

Activated c-Met is rapidly internalized in response to HGF, a step critical for downstream signaling [45, 46]. MAP4K4 depletion or the inhibition of dynamin-dependent endocytosis abrogate c-Met internalization and phosphorylation upon ligand binding and impede collagen I invasion. These data indicate that ligand-induced c-Met internalization is required for its pro-migratory function in MB, highlighting the relevance of endosomal signaling of activated GF receptors for cancer progression. The common denominator of MAP4K4 regulation of endocytic activity, Iß1 activation and c-Met endocytic signaling could be F-actin polymerization activity through Arp2/3 phosphorylation [47]. Indeed, F-actin dynamics have recently been implicated in endosomal biogenesis through the F-actin linker moesin [48], a previously identified substrate of MAP4K4 [49].

MAP4K4 depletion also prevents IR-induced migration and invasion. The impact of MAP4K4 on integrin activation and RTK activation and turnover could indicate that these processes are also relevant for irradiation-induced migration and invasion in MB. Alternatively, depletion of MAP4K4 could affect F-actin dynamics during IR-induced migration and thereby ablate the formation of invasive protrusions. This scenario is supported by the observation that depletion of MAP4K4 in UW228 cells also blocked IR–induced collagen I invasion, a treatment that did not affect endocytic turnover in these cells. However, further mechanistic insights will be necessary for the discovery of a rational approach targeting IR-induced migration.

Although depletion of MAP4K4 prevented HGF- or IR-induced migration in the two different SHH MB lines, we also observed marked differences between them. Most strikingly is the markedly higher MAP4K4 protein expression in DAOY cells. A recent proteomic study, which compared the proteome of DAOY and UW228 [26] found a number of SHH signature proteins specifically up-regulated in DAOY cells and a concomitant enrichment of key components of the SHH signaling pathway. In contrast, protein expression and signaling protein enrichment in UW228 cells indicated WNT pathway activation. However, the diverging dependence on MAP4K4 function in the two cell lines is likely due to the increased MAP4K4 expression levels in DAOY, which may be causative for its impact on the endocytic machinery. This would imply that MAP4K4 control of migration and invasion is involved in separate biological pathways, one depending on moderate MAP4K4 expression (e.g. control of F-actin polymerization, which we found in both DAOY and UW228 cells [19]) and the other - endocytic vesicle trafficking and control of integrin ß1 and c-Met receptor activation and function – on elevated MAP4K4 expression.

Thus, the identification of endocytic activity as a novel effector function of MAP4K4 for migration and invasion control opens new conceptual avenues for tailored therapeutic interventions to selectively target metastatic tumor progression independent of the underlying oncogenic alteration.

## MATERIALS AND METHODS

### Ethics approval and consent to participate

#### Human subjects

Informed consent was obtained from subjects and all research involving subject’s material was conducted under appropriate review/privacy board protocols of the Kantonale Ethikkommission Zürich (Ethics Commission of the Canton of Zürich, Switzerland). The use of patient tumour material for diagnostic and prognostic analysis was approved by the Kantonale Ethikkommission Zürich.

#### Mouse maintenance

Mouse protocols for organotypic brain slice culture were approved by the Veterinary Office of the Canton Zürich. Wild type C57BL/6JRj pregnant females were purchased from Janvier Labs and were kept in the animal facilities of the University of Zürich Laboratory Animal Center.

### Cell culture

DAOY human MB cells (ATCC, Rockville, MD, USA), UW228 [50] (provided by John Silber (Seattle, USA)) and ONS-76 [51] culture and generation of DAOY LA-EGFP and UW228 LA-EGFP cells are described in [19]. Cell lines were authenticated less than six months before initiation of experiments by SNP profiling (performed by Multiplexion, Germany). ONS-76 control cells were grown as described in [52].

### RNA expression analysis by qRT-PCR

Total RNA was isolated using RNeasy Mini Kit (Qiagen, Basel, Switzerland). 1 μg of total RNA was used as a template for reverse transcription, which was initiated by random hexamer primers. The cDNA synthesis was carried out using High capacity cDNA Reverse Transcription Kit (Applied Biosystems). qRT-PCR was performed under conditions optimized for the ABI7900HT instrument, using TaqMan^®^ Gene Expression Master Mix (4369016, Applied Biosystems). The ΔΔCT method was used to calculate the relative gene expression of each gene of interest.

### MAP4K4 gene expression analysis

Gene expression data were obtained from the R2 genomics and visualization platform (http://hgserver1.amc.nl/cgi-bin/r2/main.cgi). Gene expression levels are expressed as logical values without transformation or as the 2^nd^ log of it. The following datasets were used: Normal Brain regions - Berchtold - 172 - MAS5.0 - u133p2; Normal cerebellum - Roth - 9 - MAS5.0 - u133p2; Tumor Medulloblastoma (SHH) - Pfister - 73 - MAS5.0 - u133p2; Tumor Medulloblastoma Ependymoma - denBoer - 51 - MAS5.0 - u133p2; Tumor Medulloblastoma PLoS One - Kool - 62 - MAS5.0 - u133p2; Tumor Medulloblastoma public - Delattre - 57 - MAS5.0 - u133p2; Tumor Medulloblastoma - Gilbertson - 76 - MAS5.0 - u133p2; Tumor Glioma pediatric - Paugh - 53 - MAS5.0 - u133p2 public.

### Immunohistochemistry (IHC)

IHC of FFPE samples, of the MB Tissue Microarray (TMA) and normal brain sections was performed by Sophistolab (Muttenz, Switzerland) on a Leica BondMax instrument using Refine HRP-Kits (Leica DS9800): Epitope retrieval (ER)-solution 2 for 10 minutes at 95°C, ER-solution 2 for 20 minutes at 100°C and ER-solution 2 for 30 minutes at 100°C. The TMA slides were captured digitally using Axio Observer 2 mot plus fluorescence microscope (Zeiss, Munich, Germany). Expression was assessed blindly by an experienced pathologist at 5x to 20x magnifications and classified using H scores as high, moderate, low and negative expression.

### Immunofluorescence

Cells were processed for immunofluorescence analysis (IFA) as described in [19]. Images were acquired either on an Axioskop 2 fluorescence microscope (Zeiss) or an SP8 confocal microscope (Leica).

### Transfection and ectopic protein expression

DAOY cells were transiently transfected using Jet-Pei (101-10 Polyplus), with plasmids encoding lifeact [53] (LA)-mCherry (pLenti-LA-mCherry) and either pEGFP-C2 NIKwt (wild-type Nck-interacting kinase EGFP-NIK (murine MAP4K4)) or pEGFP-C2 NIKD152N (murine MAP4K4-kinase dead (mM4K4-k/d)) [49]. For ectopic expression of human MAP4K4, pCDNA3 vector encoding wt or K54R MAP4K4 [21] was transfected into DAOY cells as described above. Cell lines stably expressing MAP4K4-wt or MAP4K4-K54R were generated by neomycin selection of transfected cells.

### RNA interference

The cells were transfected using Dharmafect 4 transfection reagent (Dharmacon) with either Silencer Select siRNA specific for MAP4K1-5 or negative control. 30 h after transfection, the cells were re-seeded for single cell migration analysis (see 2.10). shRNA mediated depletion using Tet-inducible, shRNA-mediated MAPK4-depletion was performed as described in [19].

### MAP4K4 depletion by LentiCRISPR

Cloning of three different sgRNAs (sequence listed in supplemental materials) into BFP expressing LentiCRISPR plasmids was performed with a single-tube restriction and ligation method as described in [54]. Production of lentiviral vectors was performed according to the standard protocol. In brief, 293T cells were transfected using HEPES-buffered saline solution (HeBS) and 0.5 M calcium phosphate with LentiCRISPR, pVSV, pMDL, and pRev (latter three kindly provided by Oliver Pertz, Bern, Switzerland) in a ratio of 4.5:1.5:3:1. The media was changed after 12 h and the virus was collected at 72 h after transfection of plasmids. Viral transductions were performed using hexadimethrine bromide (H9268, Sigma-Aldrich). Depletion activity of the sgRNA sequences was tested in DAOY cells by immunoblot (IB). sgMAP4K4_0 was ineffective. The most effective sequence (sgMAP4K4_2 targeting exon 4) was chosen for further experiments.

### Live cell imaging

mCherry and EGFP fluorescence were acquired in a temperature and CO_2_-controlled SP8 Leica confocal microscope with a 63× multi-immersion objective. 60 Z-stacks of six images each were acquired (0.34 μm Z-distance, 15 s intervals, 15 min). Average intensity projections were assembled into QuickTime movies (10 fps, 150x speed). F-actin dynamics in DAOY cells stably expressing LA-EGFP [19] were recorded with a temperature- and CO_2_ controlled Zeiss Axio Observer epifluorescence microscope using a glycerol 63x objective (30 s intervals, 60 min).

### Single cell motility assay

Cells were seeded in 96-well glass bottom plates (*In Vitro* Scientific) in cell culture medium without FCS for 18 h. Cell motility was acquired thereafter -/+ HGF (20 ng/ml) using a temperature- and CO_2_-controlled ImageXpress Micro 2 microscope for 18 h. Cell speed (path length/time) was determined by manually tracking the cells at 5 min intervals for 6 - 18 h using ImageJ software (National Institutes of Health, USA).

### Spheroid invasion assay (SIA) and irradiation

1000 cells in 100 μl per well were seeded in 96 well Corning^®^ Spheroid microplate (CLS4520, Sigma-Aldrich, DAOY cells) or in cell-repellent 96 well microplate (650790, Greiner Bio-one, UW228 cells) and assay was performed and quantified exactly as described in [27]. For the Iβ1 blockage, cells were treated with 10 ug/ml Iβ1 blocking antibody or control mouse IgG for 2 h at 37°C before embedding in collagen I. Irradiation was performed using a Gulmay (Xstrahl) 200-kV x-ray unit at 1 Gy/min (kVp 200 kV; half-value layer (HVL) Z 1.03 mm Cu; Filter: 1 mm Al and 0.45 mm Cu).

### pFAK analysis in collagen I embedded cells

1’500’000 DAOY wild type, sgC or sgM4K4 cells were seeded per well in low adhesion 6 well plate CellStar^®^ (Greiner Bio-One, 657970) and incubated overnight at 37°C. Formed aggregates were embedded in 1 mL of collagen I solution and incubated 1 hour at 37°C to let the collagen polymerize. Compound treatments were as follows: C29 (5 μM, 18h), GNE-495 (1.2 μM, 18 hours) or an equivalent volume of DMSO as control -/+ HGF (20 ng/ml) at 37°C. Embedded aggregates were lysed using 500 μL of G-LYSA™ Lysis buffer (Cytoskeleton, GL36) and processed for immunoblot (IB) with FAK, pFAK and tubulin antibodies. Loading was normalized using tubulin detected on the same membrane and pFAK levels were normalized using total FAK. Relative pFAK was determined compared to solvent or compound-treated cells without HGF.

### Colony formation assay

DAOY cells were seeded at a density of 400 cells/well in a 6-well plate in complete growth medium containing 10% FBS. The cells were allowed to adhere for 24 h, and the medium was replaced with fresh complete growth medium -/+ HGF (20 ng/ml). The cells were cultured at 37°C for 10 days with medium changes every third or fourth day. The colonies were fixed in methanol for 10 min at -20°C and then stained with 0.5% crystal violet for 20 min. The number and the area of the colonies were quantified using ImageJ.

### Iß1 activation by ligand engagement (replating assay) [44]

Cells were serum starved for 24 h, detached with HyQTase and kept in suspension in serum-free medium for 1 h. Dyngo4a or C29 or a corresponding volume of DMSO were added to cells 15 min before re-plating on collagen type I (5 μg/ml) coverslips for 15 min at 37°C. Cells were subsequently fixed with 4% PFA and proceeded for IFA using a Leica SP8 confocal microscope.

### β1-integrin surface expression and activation analysis

Serum-starved DAOY cells were pre-treated with C29 (5 μM, 5 hours) or an equivalent volume of DMSO as control and detached with HyQTase, maintained in suspension for 1 hour at 37°C and seeded onto collagen-coated dishes for 15 min. 100,000 cells were then scraped into modified Tyrode’s buffer, fixed with 1 volume of 4% paraformaldehyde/1mM MgCl_2_ in PBS on ice and then incubated for 20 min on ice with anti-a-Iβ1 12G10, or anti-total Iβ1, or anti-CD44 or isotype controls. After one wash with PBS, cells were incubated with the secondary antibodies on ice for 20 min and after one wash with PBS analyzed using a BD Accuri C6 Flow Cytometer.

### Organotypic brain slice culture

Cerebellar slices were prepared from P10 C57BL/6JRj mouse pups. After 15 days in culture, sgControl and sgMAP4K4_2 tumor cell spheroids were implanted and then grown on the slices for five days as described in [36]. For HGF treatment, the feeding medium was supplemented with 20 ng/ml HGF. Purkinje cells were detected with anti-Calbindin staining and human nuclei detected with anti-human nuclei antibody. Cell dissemination was quantified as described in [27] using the human nuclei florescence with threshold settings adapted to the human nuclei signal intensities.

### Dextran uptake assay

2,000 DAOY LA-GFP cells were seeded per well in 96 well microplate μClear^®^ (Greiner Bio-One, 655090) and incubated overnight at 37°C. Medium was replaced with serum-free medium. After overnight incubation at 37°C, cells were treated with C29 (2.5 μM, 5h), EIPA (25 μM, 30 min) or an equivalent volume of DMSO as control. Starvation medium was replaced with starvation medium containing 70 kDa TMR-Dextran -/+ HGF (20 ng/ml) and the respective inhibitors, and cells were incubated at 37°C for 30 min. PFA-fixed cells were imaged using Zeiss Axio Observer microscope using a 40x objective. 25 tiles per well were acquired and used for quantification. Cell area was determined using the EGFP-channel signal. The relative dextran uptake was calculated as the TMR-Dextran integrated pixel density divided by the cell area (pixels) and compared to the control.

### c-Met internalization assay

Internalization of c-Met receptor were measured using biotinylation assay as described in [55]: Briefly, surface receptors of serum-starved sgControl or sgMAP4K4_2 DAOY cells were biotinylated using EZ-link Sulfo-NHS-LC-biotin (sulfosuccinimidyl-6-(biotin-amido) hexanoate; Thermo Scientific, Pittsburg, PA). Cells were incubated with or without HGF (20 ng/mL) for 0, 5, 15 and 30 min. After biotinylation and internalization steps, cells were lysed in RIPA buffer and biotin was precipitated from 500-800 μg of total protein using Pierce™ Streptavidin Magnetic Beads (Thermo Scientific, Pittsburg, PA). Proteins were eluted form beads with RotiLoad. Eluted proteins and total cell lysate were processed for IB.

### c-Met surface expression analysis

DAOY or ONS-76 cells were seeded in Poly-L-Lysine-coated dished and starved for 24 hours. The cells were pre-treated with C29 (5 μM, 5 hours), Dyngo 4a (5 μM, 15 minutes) or an equivalent volume of DMSO as control and then incubated with or without HGF (20 ng/ml) for 5 or 30 minutes. Cells were detached with HyQTase, resuspended into modified Tyrode’s buffer and fixed with 1 volume of 4% paraformaldehyde/1mM MgCl2 in PBS on ice for 15 min and then incubated for 20 min on ice with anti-c-Met, or anti-CD44 or isotype control. Samples were acquired using BD LSRFortessa flow cytometer (BD Bioscience). Where indicated the cells were transfected with siRNA 48 h before HGF stimulation.

### c-Met antibody feeding assay, p-c-Met internalization

DAOY LA-EGFP cells were seeded in starvation medium onto Poly-L-Lysine. 18 h later, cells were incubated with anti-c-Met antibody (1:50) for 1 h at 4°C. Cells were washed 3x with ice cold PBS and then incubated -/+ HGF for 30 min at 37°C. Cells were then fixed stained with anti-p-c-Met (1:50) and subjected to IFA.

### Cell proliferation WST-1 assay

The metabolic activity and the proliferation of the cells were determined using the WST-1 assay kit – Roche (11644807001, Sigma Aldrich) according to the manufacturer’s instructions. In brief, 2500 cells/100μl/per well (for up to 72 h incubation) and 750 cells/100μl/per well (for up to 120 h) were seeded in Greiner Bio-One μ-clear 96 well plates (655090, Greiner Bio-One) and incubated overnight at 37°C. Following appropriate incubation for each timepoint, 10 μl of the WST-1 reagent was added to each well (final concentration of WST-1 reagent per well is 1:10) and incubated at 37°C for 30 minutes. The absorbance was then measured at 440 nm.

### Statistical analysis

Unpaired student’s t-test was used to test significance of differences between samples acquired in three independent experiments. For all other analyses, one-way ANOVA repeated measures test using Bonferroni's Multiple Comparison using Prism software was performed. P-Values < 0.05 were considered significant (^*^ p ≤ 0.05, ^**^ p ≤ 0.01, ^***^ p ≤ 0.001, ^****^ p ≤ 0.0001).

## CONCLUSIONS

This study describes enhanced endocytosis of adhesion and growth factor receptors in the tumor cells as a novel pathogenic mechanism promoting receptor tyrosine kinase signaling, migration and invasion in medulloblastoma. It identified the proto-oncogenic ser/thr kinase MAP4K4, which is overexpressed in one third of MB tumors, as an important mediator of this process. Thus, MAP4K4 control of endocytic activity may represent a novel druggable mechanism to restrict metastatic dissemination of MB and of other tumors with elevated MAP4K4 expression.

## SUPPLEMENTARY MATERIALS FIGURES, TABLE AND VIDEOS









## Abbreviations

Collagen I: Collagen I
Dyn4a: Dyngo 4a
EGF: Epidermal growth factor
EIPA: Ethylisopropylamiloride
LA-EGFP: lifeact-enhanced fluorescent protein
FA: focal adhesions
F-actin: Filamentous actin
FAK: Focal adhesion kinase
Gy: Gray
HGF: Hepatocyte growth factor
i.e.: independent experiments
IR: Irradiation
k/d: kinase dead
MAP4K4: Mitogen activated kinase kinase kinase kinase 4
n.s.: Not significant
OCSCs: Organotypic cerebellum slice culture
RTK: Receptor tyrosine kinase
SgC: small guide RNA control
Wt: wild-type
