# Supplementary material for: MAP4K4 controlled integrin β1 activation and c-Met endocytosis are associated with invasive behavior of medulloblastoma cells
Source: Oncotarget. 2018 May 1;9(33):23220–36. doi: 10.18632/oncotarget.25294 (PMC5955425; doi:10.18632/oncotarget.25294)
Supplement: Supplementary file 1 [file oncotarget-09-23220-s001.pdf]

MAP4K4 controlled integrin β1 activation and c-Met endocytosis are associated with invasive behavior of medulloblastoma cells

SUPPLEMENTARY MATERIALS

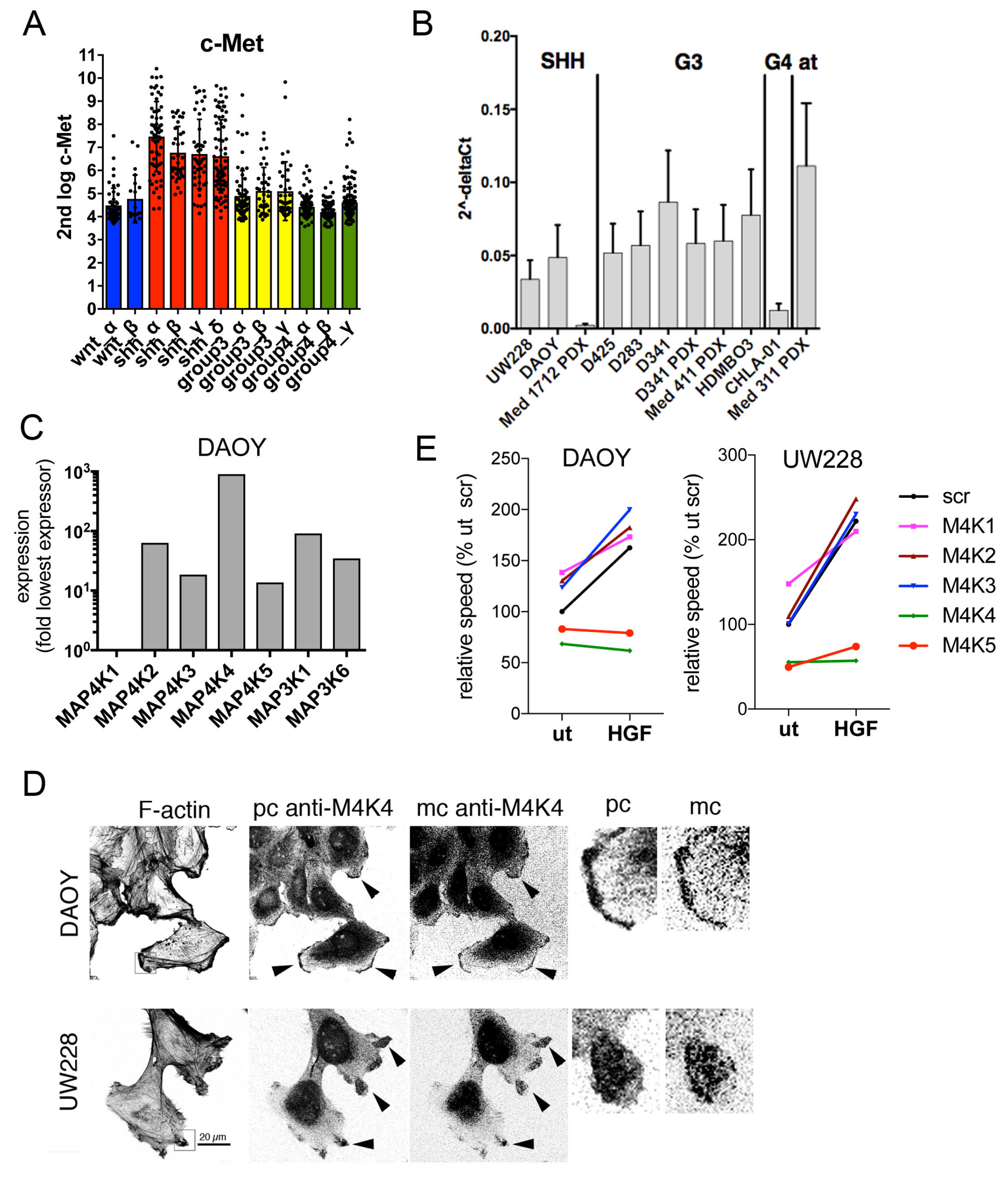

Supplementary Figure 1: (A) c-Met expression analysis in the 12 MB subtypes using data derived from the affymetrix human gene 1.1 ST array profiling of the Cavalli gene set of 763 primary MB samples. **(B)** Analysis of MAP4K4 mRNA expression in various MB cell lines (SHH: sonic hedgehog, G3: group 3, G4 group 4, at, atypical). Normalization to GAPDH mRNA. **(C)** qRT-PCR quantification of MAP4K1-5, MAP3K1 and MAP3K6 expression in DAOY cells. Normalization to GAPDH mRNA. **(D)** IFA of MAP4K4 subcellular localization in migrating DAOY and UW228 cells using both polyclonal (pc antiM4K4) and monoclonal anti-MAP4K4 (mc anti-M4K4) antibodies. Arrowheads indicate MAP4K4 localized in the lamellipodia. Magnifications are 4x of boxed areas. **(E)** Comparison of speeds (n=30) of single cells transfected with siRNAs against the indicated MAP4 kinases -/+ 20 ng/ml HGF. Table depicts p values of T-tests comparing indicated conditions -/+ HGF.

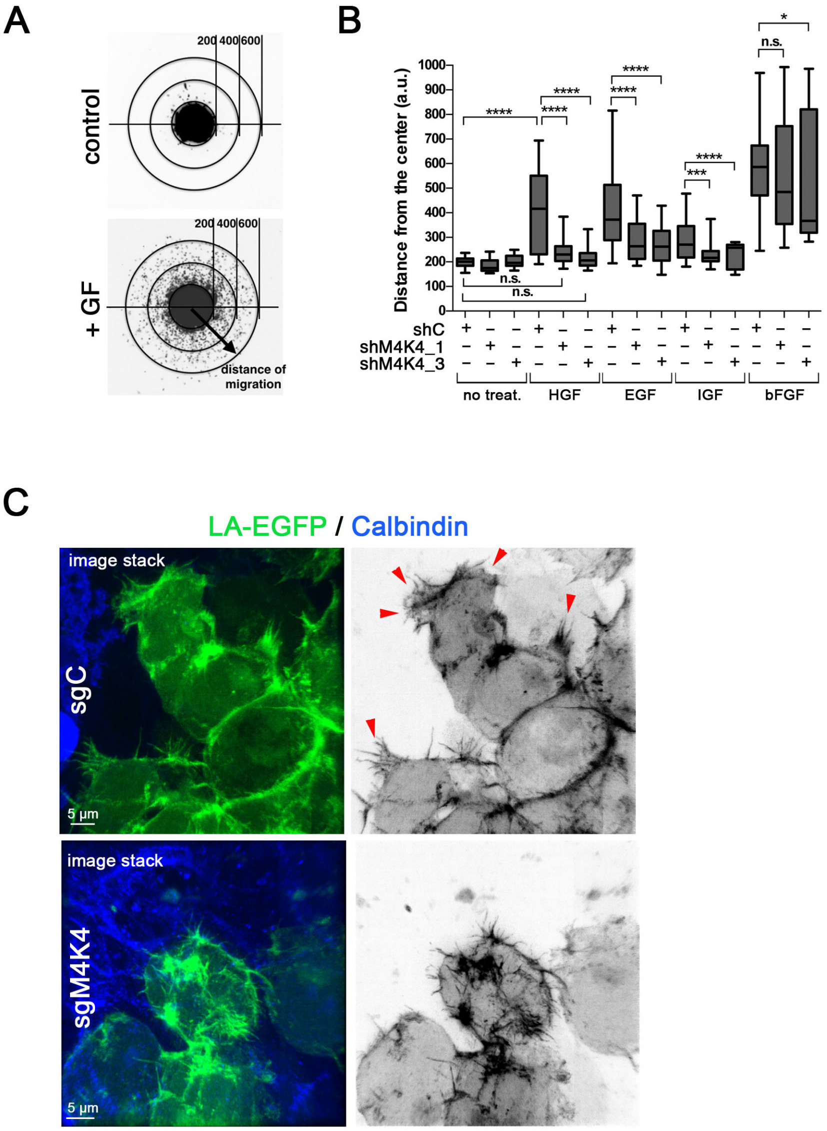

Supplementary Figure 2: **(A)** Schematic representation of Coll I invasion (spheroid invasion assay, SIA) -/+ growth factor (GF). **(B)** SIA of DAOY cells expressing control or one of two different, tetracycline-inducible MAP4K4-targeting shRNAs -/+ different GFs (HGF: 20 ng/ml, EGF: 30 ng/ml, IGF: 20 ng/ml, bFGF: 100 ng/ml). **(C)** Organotypic cerebellum slice culture with DAOY sgCtrl or DAOY sgMAP4K4_2 spheroids implanted. Projections of image stacks of individual DAOY sgCtrl or DAOY sgMAP4K4_2 cells acquired with 64x objective are shown. Inverted grey scale images represents F-actin cytoskeleton. Red arrowheads indicate invasive lamellipodia and filopodia.

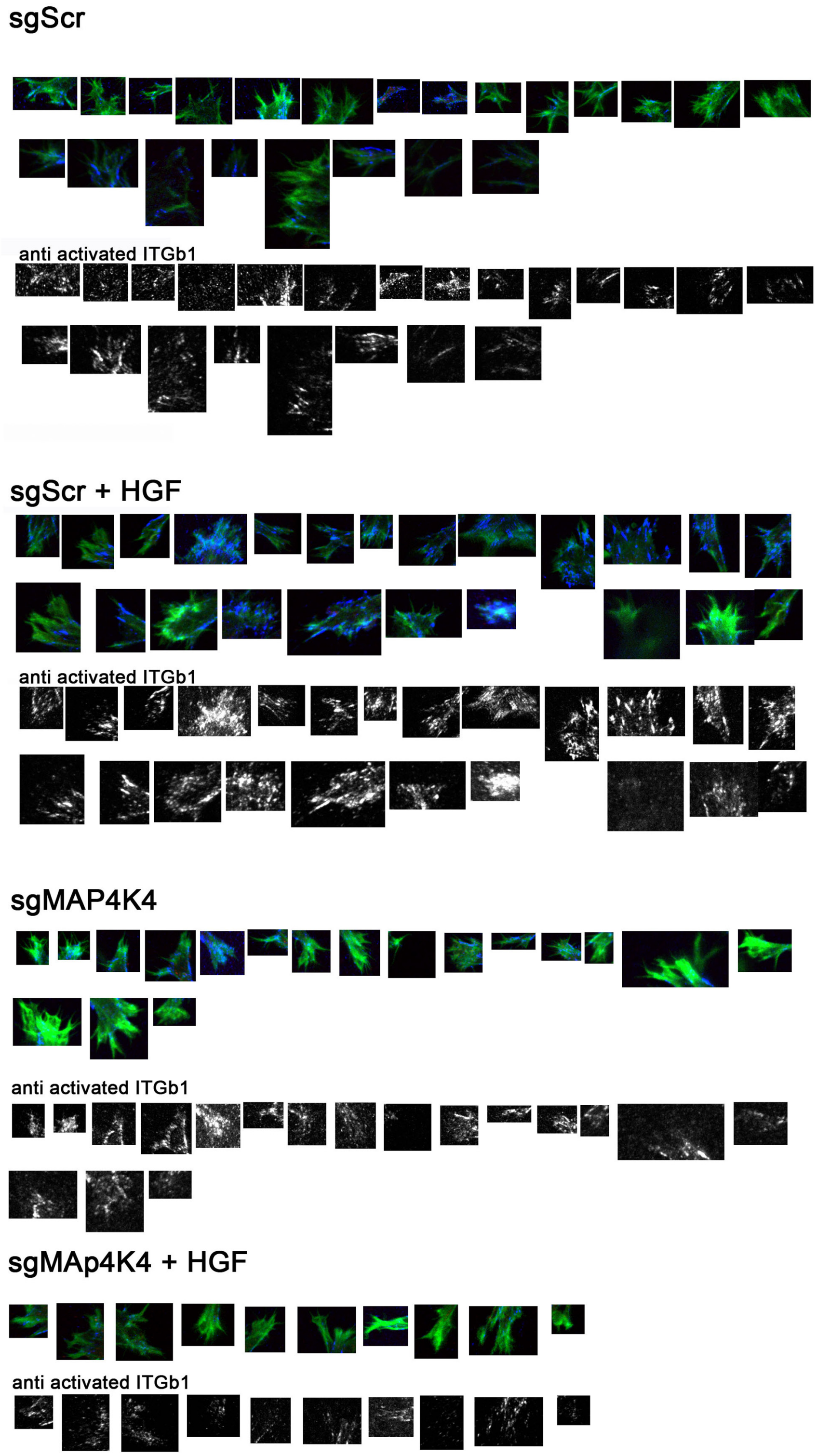

Supplementary Figure 3: Confocal microscopy images showing a-Iß1 integrin (anti-activated ITGB1, 12G10 antibody) in lamellipodia of collagen-embedded DAOY sgScr and sgMAP4K4_2 cells without or with HGF (20 ng/ml) stimulation for 24 h. Blue: Activated ß1 integrin. Green: Actin. Grey-scale images shows a-Iß1 integrin.

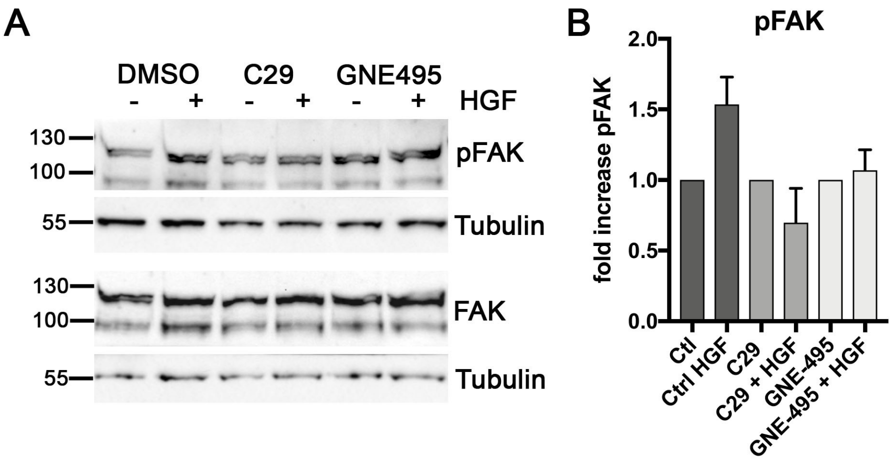

Supplementary Figure 4: Analysis of pFAK in collagen I-embedded DAOY cells -/+ C29 (5 µM, 18h) or GNE-495 (1.2 µM, 18h) and -/+ HGF (20 ng/ml). **(B)** Quantification of relative pFAK after normalization with tubulin and FAK (n = 2 independent experiments).

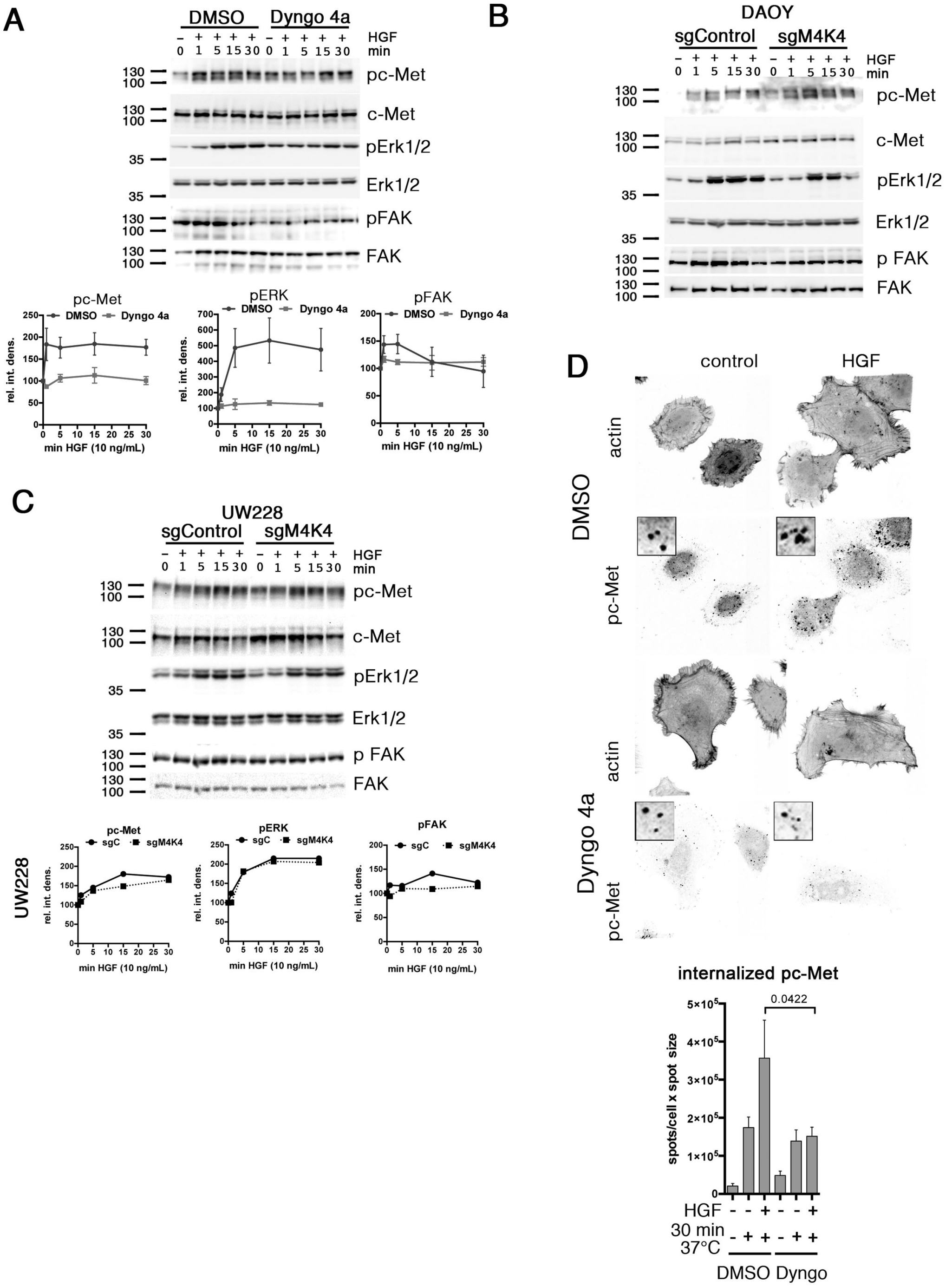

Supplementary Figure 5: (A) Top: IB analysis of c-Met, Erk1/2 and FAK phosphorylation in response to HGF stimulation -/+10 µM Dyngo 4a. Bottom: XY line plots of mean integrated pixel densities and SEM of phospho bands from 3 experiments against time in min after HGF stimulation. **(B)** IB analysis of c-Met, Erk1/2 and FAK phosphorylation in response to HGF stimulation in DAOY sgC and sgMAP4K4_2 cells. **(C)** Top: IB analysis of c-Met, Erk1/2 and FAK phosphorylation in response to HGF stimulation in UW228 sgC and sgMAP4K4_2 cells. Bottom: XY line plots of mean integrated pixel densities and SEM of phospho bands from 3 experiments against time in min after HGF stimulation. **(D)** Upper: confocal IFA of PLL adhering DMSO or Dyn4a-treated cells. pc-Met signal after raising temperature from 0° to 37°C -/+ HGF. Insets are 4x magnifications. Bottom: bar diagram of single cell analyses of (spots/cell * size of spots (n=13, mean and SD).

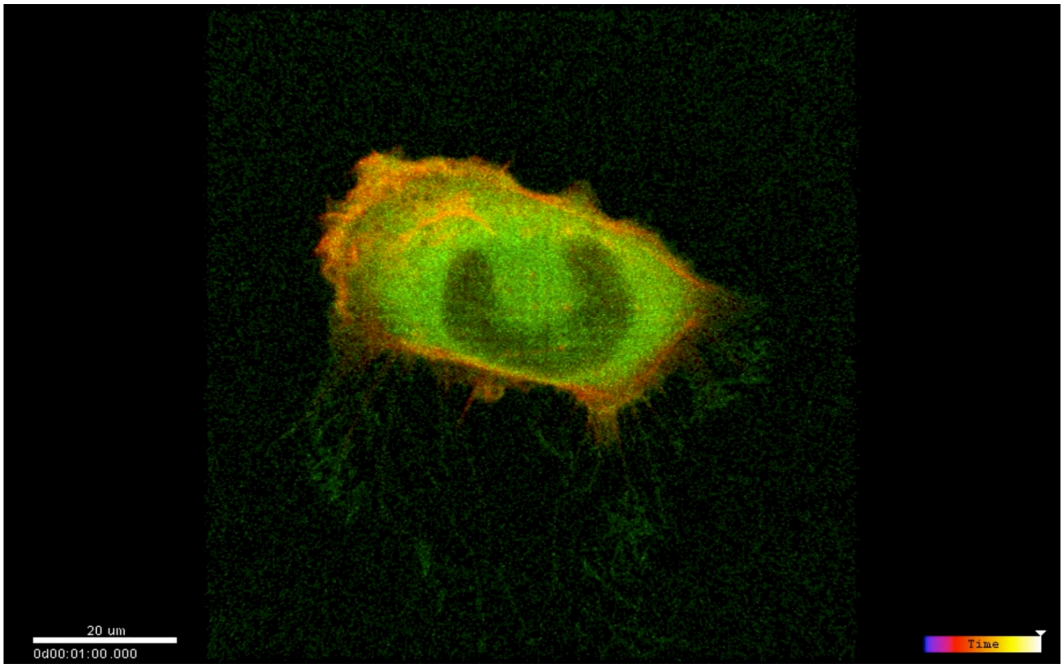

Supplementary Movie 1: Time-lapse imaging of DAOY cells transiently transfected with LA-mCherry (red) and EGFP-NIKwt (green).

See Supplementary File 1

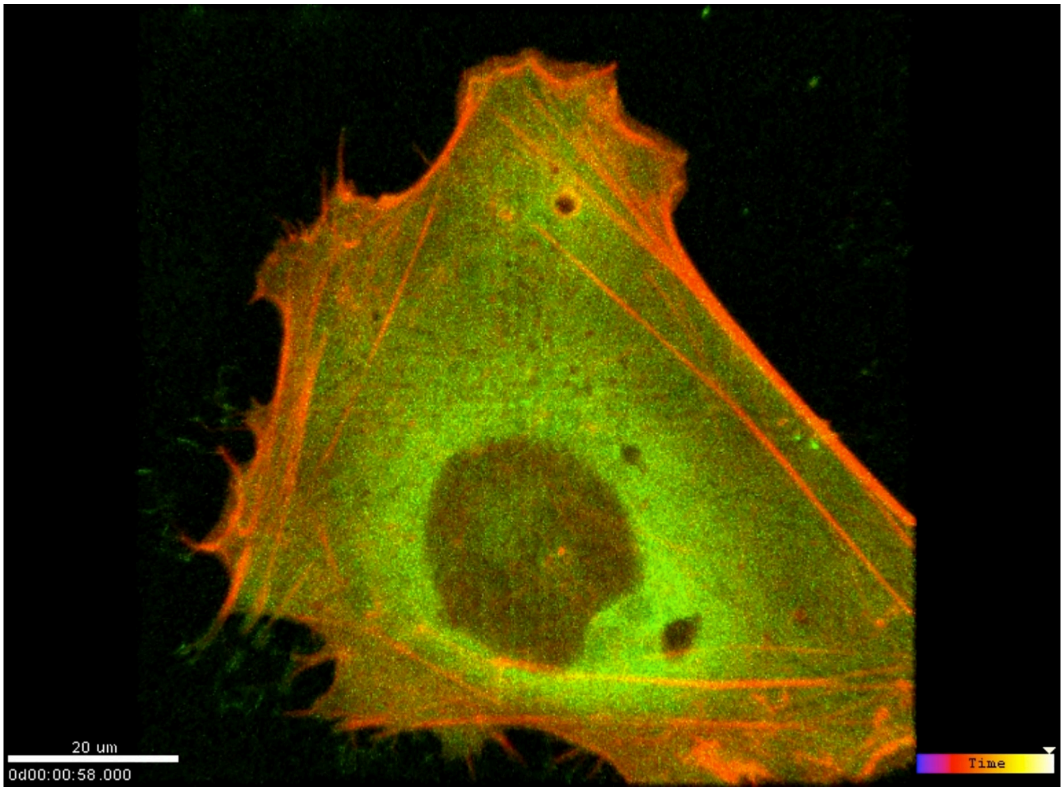

Supplementary Movie 2: Time-lapse imaging of DAOY cells transiently transfected with LA-mCherry (red) and EGFP-NIKD152N (green).

See Supplementary File 2

Reagents list:

See Supplementary File 3
