# Supplementary material for: MAP4K4 controlled integrin β1 activation and c-Met endocytosis are associated with invasive behavior of medulloblastoma cells
Source: Oncotarget. 2018 May 1;9(33):23220–36. doi: 10.18632/oncotarget.25294 (PMC5955425; doi:10.18632/oncotarget.25294)
Supplement: Supplementary file 4 [file oncotarget-09-23220-s004.docx]

**Supplemental materials**

**MAP4K4 controlled integrin β1 activation and c-Met endocytosis are associated with**

**invasive behavior of medulloblastoma cells**

**Reagents list:**

| Reagent name | Dilution from stock | Company |
| --- | --- | --- |
| Rabbit pc anti-MAP4K4 (IFA) | 1:100 | Bethyl, A301-502A |
| Mouse mc anti-MAP4K4 (IFA) | 1:100 | Abnova , MO-7, clone 4A5 |
| Rabbit pc anti-MAP4K4 (IHC) | 1:50 | Atlas, HPA008476 |
| Rabbit pc anti-MAP4K4 (IB) | 1:1000 | Abcam, 80418 |
| Rat mc anti-c-Met (PE) | 1:100 | eBioclone 7, ebioscienence, 12-8854-80 |
| PE Rat IgG1, κ Isotype Ctrl for c-Met | 1:100 | Biolegend, 400407 |
| pc-phospho c-met (phospho Tyr1230/Tyr1234/Tyr1235) | 1:50 | Merck, 07-810 |
| mc anti-Iβ1 (12G10), active Iβ1 | 1:200 | Abcam. ab30394 |
| Anti-total Iβ1-FITC, mouse IgG2a | 1:200 (FACS) | BD Biosciences, 555573 |
| Iβ1 blocking antibody | 10 µg/ml | Abcam, ab24693 |
| Mouse IgG isotype control | 1:100 | EBioscieces, 164714-85 |
| Anti-CD44-AlexaFluor 488 | 1:100 | BioLegend, 103016 |
| IgG2a Isotype control for CD44-AlexaFluor 488 | 1:100 | BioLegend, 400625 |
| Rabbit mc anti-Rab-7 | 1:200 | Cell signaling, 9367 |
| Cy5-conjugated anti-Rat Anti-Mouse IgG | 1:300 | Jackson Immunoresearch, 415-175-166 |
| Cy3-conjugated Donkey anti-Rabbit IgG (H+L) | 1:300 | 711-165-152 and, Jackson Immunoresearch |
| Control mouse IgG | 10 µg/ml | Santa Cruz sc-2025 |
| anti-MAP4K4 | 1:1000 | Abcam, 80418 |
| anti-ERK1/2 | 1:1000 | Cell signaling, 9102 |
| anti-phospho-ERK1/2 | 1:1000 | Cell signaling, 9101 |
| Anti-FAK | 1:1000 | 610088, BD Biosciences |
| Anti-pFAK | 1:1000 | 44624G, Life Technologies |
| anti-c-Met (IB, IFA) | 1:1000 | Cell signaling, 8198 |
| anti-c-Met (ab feeding) | 1:50 | Cell Signaling, 11C4, 56315 |
| anti-phospho-c-Met | 1:1000 (IB), 1:150 (IFA) | Merck Milipore, 07-810 |
| anti-tubulin | 1:1000 | Sigma Aldrich, T9026 |
| anti-human nuclei | 1:700 | Merck Millipore MAB4383 |
| Hoechst | 1:5000 | Sigma-Aldrich, B2883 |
| phalloidin-488 | 1:500 | Molecular Probes |
| HyQTase |  | HyClone, SV30030.0 |
| Glycergel |  | Dako, C0563 |
| Dextran Tetramethylrhodamine | 1 mg/ml | ThermoFischer, D1818 |
| EZ-Link Sulfo-NHS-Biotin | 0.5 mg/ml | ThermoFischer, 21331 |
| Dynasore  Dyngo 4a | 20 µM  5 µM | Sigma Aldrich, SML0340 |
| EIPA | 25 µM | Sigma Aldrich, A3085 |
| C29 | 0.5 µM SIAs, long term  2.5 µM, short term | Genentech |
| GNE-495 | 1.2 µM | Genentech |
| HGF | 20 ng/ml | Preprotech |
| MeSNa (Sodium-2-mercaptoethanesulfonate) |  | Sigma-Aldrich, 63705 |
| MeSNa Buffer | 100 mM NaCl, 50 mM Tris–HCl, pH 8.6, 1 mM MgCl_2_, 1 mM CaCl_2_ |  |
| Poly-L-Lysine |  | Sigma Aldrich, PLL, P9155 |
| Biotinylation buffer | 0.154 M NaCl, 10 mM Hepes, pH 7.6, 3 mM KCl, 1 mM MgCl_2_, 1 mM CaCl_2_, 10 mM glucose |  |
| streptavidin beads |  | ThermoFischer, 88816 |
| RotiLoad |  | KarlRoth, K929.1 |

| siRNA, sgRNA, PCR primers | Working concentration | Company |
| --- | --- | --- |
| Silencer select MAP4K1 | 5 nM | Ambion, 22080 |
| Silencer select MAP4K2 | 5 nM | Ambion, 1688 |
| Silencer select MAP4K3 | 5 nM | Ambion, S552 |
| Silencer select MAP4K4 | 5 nM | Ambion, 18095 |
| Silencer select MAP4K5 | 5 nM | Ambion, 22078 |
| Silencer select MAP3K1 | 5 nM | Ambion, S8668 |
| Silencer select MAP3K6 | 5 nM | Ambion, S17289 |
| Silencer select control | 5 nM | Ambion,4390843 |
| sgMAP4K4_0 (exon 6) | TGTGATTCACCGGGATATCAAGG |  |
| sgMAP4K4_1 (exon 7) | GGGCGGAGAAATACGTTCATAGG |  |
| sgMAP4K4_2 (exon 4) | CAGGACATGATGACCAACTCTGG |  |
| MAP4K1 primer pairs |  | Applied Biosystems, Hs00179345_m1 |
| MAP4K2 primer pairs |  | Applied Biosystems, Hs00387564_m1 |
| MAP4K3 primer pairs |  | Applied Biosystems, Hs00269284_m1 |
| MAP4K4 primer pairs |  | Applied Biosystems, Hs00377415_m1 |
| MAP4K5 primer pairs |  | Applied Biosystems, Hs00179088_m1 |
| MAP3K1 primer pairs |  | Applied Biosystems, Hs00394890_m1 |
| MAP3K6 primer pairs |  | Applied Biosystems, Hs01085775_m1 |
| GAPDH |  | Applied Biosystems, Hs02786624_g1 |
| EGFP-MAP4K4-wt | Murine MAP4K4 (NIK) | Baumgartner et al. PNAS 2006, 103:13391-13396 |
| EGFP-MAP4K4-k/d (D152N) | Murine MAP4K4 (NIK) | Baumgartner et al. PNAS 2006, 103:13391-13396 |
| MAP4K4-wt | Human MAP4K4 | Wright et al. MCB 2003, 23:2068-2082 |
| MAP4K4-k/d (K54R) | Human MAP4K4 | Wright et al. MCB 2003, 23:2068-2082 |
|  |  |  |
| Modified Tyrode’s buffer | 137 mM NaCl, 2.7 mM KCl, 12 mM NaHCO3, 5 mM HEPES, 1 mM MgCl2, 0.5 mM CaCl2, 0.1% glucose, pH 7.4 |  |
